# Evidence on SGLT2 Inhibitors’ Efficacy in Older and Frail Patients

**DOI:** 10.3390/jcm15062219

**Published:** 2026-03-14

**Authors:** Anna Kochanowska, Artur Mamcarz, Marcin Wełnicki

**Affiliations:** 3rd Department of Internal Medicine and Cardiology, Medical University of Warsaw, 04-749 Warsaw, Poland

**Keywords:** SGLT2 inhibitors, older adults, frailty, efficacy

## Abstract

Sodium-glucose cotransporter 2 (SGLT2) inhibitors have proven their favorable cardiovascular and nephroprotective benefits in large randomized-controlled trials (RCTs). Given that older adults constitute a substantial part of patients with type 2 diabetes mellitus (T2DM), heart failure (HF), and chronic kidney disease (CKD), they are the primary target population for SGLT2 inhibitor therapy. However, their representation in clinical trials remains low and it does not reflect the real-life heterogeneity of this group of patients. As chronological age alone does not adequately reflect the biological age, it is important to evaluate older adults using a multidimensional approach, particularly with regard to frailty. This review aims to summarize and critically appraise the available evidence regarding the efficacy of SGLT2 inhibitors in older and frail adults, with a focus on age-specific outcomes, such as cognitive outcomes, risk of sarcopenia, functional activity and current gaps in evidence related to frailty.

## 1. Introduction

The global population is aging rapidly, and the prevalence of chronic conditions such as type 2 diabetes mellitus (T2DM), heart failure (HF), and chronic kidney disease (CKD) continues to rise. With advancing age, the prevalence of frailty, a multidimensional syndrome characterized by diminished physiological reserves and increased vulnerability to stressors, markedly increases, further complicating the management of chronic diseases in this population.

Sodium-glucose cotransporter 2 (SGLT2) inhibitors have proven their favorable cardiovascular and nephroprotective benefits in large randomized-controlled trials (RCTs). Given that older adults constitute a substantial part of patients with T2DM, HF, and CKD, they may be viewed as the primary target population for SGLT2 inhibitor therapy. Therefore, understanding the efficacy of SGLT2 inhibitors in frail patients is essential. Despite this, their representation in randomized trials remains limited, and real-world utilization of SGLT2 inhibitors in this group is suboptimal. Several post hoc analyses of RCTs have provided valuable insights into the subgroup of elderly and frail patients. While they may provide valuable insight and raise new questions for further studies, they are prone to various limitations, including reduced statistical power, selection bias, increased possibility of type I errors, and lack of prespecified baseline characteristics or endpoints relevant to older adults [1].

Because frailty domains are not routinely assessed in clinical trials, many studies lack crucial information on participants’ functional and cognitive status. This, alongside the absence of a unified frailty definition or scoring system, is one of the underlying reasons why conducting meta-analyses in this area remains challenging [2]. One way to operationalize frailty retrospectively is through the cumulative deficit approach proposed by Rockwood and Mitnitski [3]. Although not without limitations, it has been shown to reliably predict adverse outcomes. This predictive validity was confirmed in post hoc analyses of the CANVAS and CREDENCE trials [4].

Moreover, there is a paucity of clinical trials specifically designed for these populations, and the few available are predominantly observational. Some meta-analyses summarized findings across diverse patient cohorts (Table 1) [2,5,6,7,8,9,10,11], but they often focus on broad age categories and do not address domains relevant to the care of older individuals.

Therefore, this review aims to integrate and critically appraise the available evidence on the efficacy of SGLT2 inhibitors in older and frail adults. It first discusses their metabolic, cardiovascular and renal effects, with particular emphasis on outcomes in older populations, and then examines geriatric domains such as cognitive health, body weight and composition, and the impact on frailty. Furthermore, it identifies important gaps in the current evidence base, particularly regarding underrepresented populations such as very old, malnourished, and institutionalized patients. This review attempts to bridge the gap between age-based subgroup analyses and frailty-oriented interpretation of SGLT2 inhibitor efficacy, two perspectives that have been rarely examined together in reviews.

## 2. Efficacy of SGLT2 Inhibitors

### 2.1. Metabolic Outcomes

In older diabetic patients, SLGT2 inhibitors significantly reduce the glycated hemoglobin (HbA1c) concentration [9,12,13]. This effect is diminished with increasing age (0.24%; 95% confidence interval (CI), 0.10% to 0.38%, less HbA1c lowering per 30-year higher age in SGLT2 inhibitors monotherapy) [6] and frailty (0.08%; 95%CI 0.02% to 0.14%, *p* = 0.029 smaller reduction per 0.1-point increase in the frailty index) [2]. In a meta-analysis of trials in older patients with type 2 diabetes mellitus (T2DM) and heart failure (HF), SGLT2 inhibitors did not significantly reduce HbA1c compared to non-SGLT2i treatments (*p* = 0.34). However, sensitivity analysis excluding a low-dose canagliflozin trial revealed a modest but significant HbA1c reduction (*p* = 0.009) [7]. More importantly for the older population, SGLT2 inhibitors did not increase the risk of hypoglycemic events [5,10,12,13,14] and several RCTs even demonstrated a beneficial effect compared to a placebo [15]. In patients with a very complex health status, the American Diabetes Association (ADA) even recommended avoiding reliance on HbA1c and focusing on minimizing the risk of hypoglycemia and symptomatic hyperglycemia as a reasonable treatment goal [16].

In the EMPA-ELDERLY study, a randomized, double-blind, placebo-controlled trial in elderly patients, SGLT2 inhibitors significantly reduced body weight (placebo-adjusted mean change: −2.37 kg; 95%CI −3.07 to −1.68; *p* < 0.001), fat mass and body water. The study also showed a trend towards a reduction in muscle mass, skeletal muscle mass, lean body mass, bone mineral content and handgrip strength; however, these findings did not reach statistical significance [12]. This contrasts with the results of the previous meta-analysis, where SGLT2 inhibitors significantly reduced weight-, fat- and muscle-related changes [17]. However, this meta-analysis was conducted in the general adult population, and the mean age in most of the included studies was below 65 years. On the other hand, one of the inclusion criteria in the EMPA-ELDERLY study was body mass index (BMI) ≥22 kg/m^2^, which raises concerns about the representativeness and generalizability of its findings to the broader older population.

### 2.2. Cardiovascular Outcomes

The cardiovascular efficacy of SGLT2 inhibitors in older adults has been confirmed by several meta-analyses. The most consistent effect was observed for hospitalization for HF, with a significant reduction across all subgroups, including different disease types, study designs, and age categories [5,7,9,18]. This reduction ranged from 28% to 34% among patients aged ≥ 65 years. A similar benefit was observed in patients aged ≥ 75 years, with no significant interaction detected [5]. These findings have also been supported by real-world data from elderly populations with acute decompensated HF (ADHF). In the OASIS-HF study, which included patients aged ≥75 years with ADHF, long-term SGLT2 inhibitor therapy was associated with a lower incidence of the composite outcome of cardiovascular death and HF rehospitalization, fewer annual HF rehospitalizations (0.22 ± 0.13 vs. 0.14 ± 0.08 visits per year; *p* = 0.019), and a slower decline in estimated glomerular filtration rate (eGFR) compared to conventional therapy [19]. Reduction in HF hospitalizations is particularly relevant in older adults. It has important implications not only for cardiovascular outcomes but also for maintaining functional independence and cognitive health.

Other cardiovascular outcomes varied across meta-analyses and patient populations, though overall trends remained favorable. The risk of all-cause mortality in the elderly population was reduced by 12–19%, with the greatest improvement in older patients with chronic kidney disease (CKD; 20%) and HF (10–19%), and little to no significant benefit in those with T2DM. Similarly, the risk of cardiovascular death (CVD) was decreased by 18–20% in the overall elderly population, although this effect varied across disease subgroups [5,7,9]. Among large studies, the most pronounced mortality benefits were demonstrated in the EMPA-REG OUTCOME trial, an empagliflozin cardiovascular-outcome trial (CVOT) [20]. Interestingly, in a meta-analysis conducted by Aldafas et al., no significant change in all-cause mortality was observed in RCTs alone [risk ratio (RR) 0.90; 95% CI 0.87 to 1.05]. However, when observational studies were included, the effect reached statistical significance [7]. Real-world studies often include a higher proportion of patients with greater comorbidity and higher baseline cardiovascular risk compared to RCTs, which may account for the more pronounced effects observed in such studies.

In a network meta-analysis using individual participant data, SGLT2 inhibitors were associated with a greater reduction in risk of major adverse cardiovascular events (MACEs) in older compared to younger patients with diabetes (hazard ratio (HR) 0.76; 95% CI 0.62 to 0.93 per 30-year age increase), despite smaller reductions in HbA1c [6]. Furthermore, the number needed to treat (NNT) declined with increasing age [9]. However, in other meta-analyses, SGLT2 inhibitors reduced the risk of MACEs in patients with HF or chronic kidney disease (CKD), but not in patients with T2DM and atherosclerotic cardiovascular disease (ASCVD) [5,7,9]. This heterogeneity may result from various trial designs, population characteristics and specific SGLT2 inhibitors used. It is worth noting that there was a significant association between individual drugs and MACEs (*p* = 0.05), with empagliflozin being the only drug significantly reducing the risk of MACEs in elderly patients [5,9]. This interaction may be explained by several hypotheses, such as higher SGLT2/SGLT1 selectivity of empagliflozin or differences in study populations.

SGLT2 inhibitors have also been shown to significantly reduce blood pressure (BP) in the elderly population, significantly lowering systolic BP (SBP) by a mean difference (MD) of −4.45 mmHg (95% CI −6.64 to −2.25) and diastolic BP (DBP) by −1.17 mmHg (95%CI −2.07 to −0.26) [9]. A post hoc analysis of the SACRA study found that adding empagliflozin to angiotensin receptor blockers (ARBs) and other antihypertensive medications in elderly diabetic patients (aged ≥75 years) with uncontrolled nocturnal hypertension was associated with significant reductions in 24 h ambulatory BP (ABPM; −8.7 ± 2.6 mm Hg) and daytime SBP (−10.8 ± 2.8 mm Hg) [21].

Another important cardiovascular outcome that requires highlighting is the anti-arrhythmic potential of SGLT2 inhibitors. Although the evidence in the general population is well-established, it remains scarce in older and frail adults. A recent meta-analysis evaluating the ability of SGLT2 inhibitors to prevent atrial fibrillation (AF) demonstrated their protective role against AF in the overall population [22]. However, subgroup analyses revealed that the results may vary depending on the clinical setting. Unfortunately, the analysis was not performed according to the frailty level or the age groups. Existing evidence comes mainly from observational studies. In a large-cohort study using propensity score matching, the risk of new-onset AF in older adults (aged ≥ 66 years) was significantly lower in the SGLT2 inhibitor group compared to the dipeptidyl peptidase 4 inhibitor (DPP-4i) or glucagon-like peptide 1 receptor agonist (GLP-1RA) groups [23]. Moreover, their beneficial effects were observed not only in AF prevention, but also in the reduction in arrhythmia recurrence. An up-to-date prospective cohort study in patients aged ≥65 with persistent AF also demonstrated significantly better outcomes in patients treated with an SGLT2 inhibitor in addition to standard AF therapy, compared with standard treatment alone [24]. The benefits included not only the arrhythmia recurrence, but also left atrial diameter, myocardium fibrosis biomarkers and very important geriatric domains such as mobility and functional activity. However, the mean age in the aforementioned study was relatively low (68.4 and 69.4 in the research and control groups, respectively), which highlights the need for further studies in even older populations.

### 2.3. Renal Outcomes

The definitions of renal or cardiorenal composite outcomes were generally similar, though they varied slightly across studies. SGLT2 inhibitors consistently reduced the cardiorenal risk by 23–49% in all age subgroups, using cutoffs of either 65 or 75 years [5,9,11]. These findings were further supported by observational studies, where the observed effect size was even more pronounced [18,25]. Although the relative benefits in slowing kidney disease progression were consistent across age groups [26,27], patients older than 70 years exhibited a lower incidence of kidney events and a slower baseline rate of eGFR decline. As a result, the absolute renal benefit in this age group was smaller and the NNT higher [27]. However, older adults typically have a higher baseline cardiovascular risk, which likely amplifies the observed cardiovascular benefits of SGLT2 inhibitors in this population. Karagiannis et al. also observed a reduced risk of developing albuminuria (RR 0.71; 95% CI 0.59 to 0.85) and acute kidney injury (AKI) (RR 0.70; 95% CI 0.53 to 0.92) in elderly patients with T2DM [11]. In a large-cohort study including 139,607 older patients, initiation of an SGLT2 inhibitor was associated with a reduced risk of AKI compared to initiation of a DPP-4i or a GLP-1RA [28]. Moreover, Shah et al. reported a trend toward reduced AKI risk, but the association did not reach statistical significance (RR 0.85; 95% CI 0.71 to 1.01; *p* = 0.07) [5].

### 2.4. Outcomes in Frail Patients

Evidence on the efficacy of SGLT2 inhibitors in frail patients remains limited and fragmented. To date, no RCTs have been specifically designed to assess outcomes in frail individuals. Available data largely stem from post hoc analyses of large RCTs, in which frailty was retrospectively assessed, mostly using frailty indices (FIs) based on the Rockwood cumulative-deficit approach [4,29,30,31] (Table 2). In all these studies, the beneficial effects of SGLT2 inhibitors on primary outcomes remained consistent across all frailty strata, suggesting that frailty status does not diminish the clinical efficacy of these agents. However, some nuances are worth highlighting. In the DELIVER trial, patients with higher levels of frailty experienced greater improvement in symptoms, physical function, and quality of life [30]. In the EMPEROR-Preserved trial, empagliflozin was associated with improvement in frailty status over the course of follow-up [29]. It is noteworthy that in the EMPA-KIDNEY trial, patients with the highest level of frailty (based on the risk of hospitalization) were less adherent and more likely to discontinue the treatment [32]. Another factor that might influence the results of post hoc analyses of large randomized trials was that the level of frailty was consistently associated with higher baseline BMI [4,29,30,31]. Some of the studies almost entirely excluded malnourished and underweight patients [4]. This raises concerns about their applicability to frail patients at the anorexic–malnourished end of the phenotype spectrum.

To address the scarcity of evidence, one individual-patient data (IPD) meta-analysis attempted to evaluate the effects of SGLT2 inhibitors in frail populations [2]. However, due to insufficient data on function or comorbidity enabling the calculation of FI, it excluded trials primarily focused on cardiovascular outcomes. This resulted in limited statistical power due to the small number of relevant endpoints.

Additional insights come from retrospective observational studies using propensity score matching to compare SGLT2 inhibitors with other oral glucose-lowering drugs (oGLDs). These studies showed that SGLT2 inhibitors were more effective than DPP-4 inhibitors and had comparable efficacy to GLP-1 RAs across most outcomes [33,34]. However, in terms of dialysis or renal transplant risk, SGLT2 inhibitors demonstrated superiority over GLP-1 RAs [34]. Furthermore, a few prospective studies focusing on frailty-related domains such as cognitive performance and physical function have been published [35,36]. In frail patients with hypertension and diabetes, a 3-month follow-up revealed a significant improvement in cognitive function and 5 m gait speed with empagliflozin compared to in the control group [35]. Similar results were found in frail patients with HF with preserved ejection fraction (HFpEF), where empagliflozin, unlike insulin or metformin, improved cognitive function. Additionally, empagliflozin showed beneficial effects on physical impairment compared to insulin [36].

Although these findings are valuable, they highlight the urgent need for prospective trials explicitly targeting frail older adults and frailty assessment at baseline in all future cardiovascular trials that include older people.

### 2.5. Cognitive Outcomes

The potential impact of SGLT2 inhibitors on cognitive function has recently gained increasing scientific interest. Although research in this area is still limited, emerging evidence suggests a potential role of SGLT2 inhibitors in preserving and improving brain function in older adults. A meta-analysis of 12 studies in diabetic patients has shown that SGLT2 inhibitor users had a significantly lower dementia incidence compared to non-users (HR = 0.68, 95% CI: 0.50 to 0.92), especially in individuals aged ≥ 60 years [37]. Similar results were demonstrated in a recent meta-analysis of five cohort studies (RR = 0.77; 95% CI: 0.71 to 0.84, *p* < 0.01) [38]. The evidence on the cognitive effects of SGLT2 inhibitors is mixed and appears to depend on baseline cognitive status. Several RCTs and prospective cohort studies have demonstrated significant cognitive improvements in older adults with mild cognitive impairment (MCI) or dementia [35,36,39]. However, no such benefits were observed in individuals with normal baseline cognitive function, which may be due to the smaller sample size, younger age of the participants, and insufficient follow-up duration in these studies [37,40]. Another limitation is the pooled analysis of dementia, behind which lies a spectrum of underlying diseases. The evidence of the role of SGLT2 inhibitors in Alzheimer’s disease (AD) is scarce. However, in a recently published Mendelian randomization study, SGLT2 inhibition was associated with a lower likelihood of developing AD for every 1 SD decrease in HbA1c (OR  =  0.48, [0.36, 0.63], *p*  <  0.001) [41]. Mendelian randomization was also used to assess the link between SGLT2 inhibition and cerebral small-vessel disease. In this study, genetically predicted SGLT2 inhibition was associated with a lower risk of small-vessel stroke, microbleeds, and improved integrity of white-matter microstructure [42].

While improvements in cognitive outcomes may be partially attributed to the modulation of stroke risk factors by SGLT2 inhibitors, such as better glycemic control, improved lipid profile, and reduced adiposity, emerging evidence also points to direct effects on the central nervous system that may contribute independently to cognitive benefits. SGLT2 inhibitors have been shown to improve brain insulin sensitivity and mitochondrial function, as well as reduce neuroinflammation, apoptosis, and oxidative stress [35,43]. Additional mechanisms that may influence dementia risk include decreased accumulation of beta-amyloid plaques [44] and inhibition of mechanistic target of rapamycin (mTOR) hyperactivation [45], although a more in-depth discussion of these molecular mechanisms is beyond the scope of this review [46,47].

## 3. Limitations of Available Studies

### 3.1. Underrepresentation of Older Patients in Large Trials

According to International Council for Harmonisation of Technical Requirements for Pharmaceuticals for Human Use (ICH) guidelines from 1993, new drugs should be evaluated across all age groups, and populations likely to receive the drug in clinical practice should be reasonably represented in clinical trials [48]. Many trials still fail to include a satisfactory number of older patients, especially aged ≥ 75 years (Table 3). In the early CVOT, such as EMPA-REG OUTCOME and DECLARE-TIMI 58, very old adults (≥75 years) constituted 9% and 6%, respectively [15,20]. Higher representation of older adults has been observed in more-recent studies, probably not merely due to publication timing, but because of the clinical conditions they targeted. For instance, HFpEF is a disease predominantly affecting older people, while transcatheter aortic valve implantation (TAVI) is a preferred method of severe aortic stenosis treatment in this population. Consequently, trials such as EMPEROR-Preserved and DELIVER included over 40% of participants aged ≥75 years, while the DAPA-TAVI study enrolled over 70% of patients aged above 80 [49,50,51].

### 3.2. Exclusion of Underweight and Malnourished Patients

Frailty exists along a spectrum, from a sarcopenic–obese phenotype, characterized by obesity, insulin resistance, and high cardiovascular risk, to an anorexic–malnourished phenotype, marked by weight loss, reduced insulin resistance, and fewer cardiovascular risk factors. This metabolic heterogeneity may have clinical implications, suggesting that not all frail older individuals may profit from SGLT2 inhibitor treatment to the same extent [56]. Patients with a sarcopenic–obese frailty phenotype, due to increased baseline cardiovascular risk, may experience greater absolute risk reduction, and consequently a lower NNT, from SGLT2 inhibitor therapy compared to non-frail individuals. In contrast, underweight, frail patients may have a higher mortality risk related to malnutrition rather than cardiovascular risk, and the benefits of such treatment in this group might be limited. Further weight loss associated with SGLT2 treatment may also increase the risk of adverse events, such as dehydration, hypotension, falls, and fractures. Some experts suggested that extremely anorexic frail patients will not benefit from newer antidiabetic therapies [56]; however, we desperately need high-quality evidence to confirm or reject this plausible hypothesis.

In post hoc analyses of RCTs, a higher level of frailty was significantly associated with higher baseline BMI. In the DAPA-HF trial, mean BMI among patients with the highest degree of frailty was 30.6 ± 6.1 kg/m^2^; similarly, in DELIVER—32.1 ± 6.2 kg/m^2^—in EMPEROR-Preserved—32.7 ± 6.1 kg/m^2^—and in EMPA-Kidney—32.1 ± 7.1 kg/m^2^ [29,30,31,32]. In the post hoc analysis of CANVAS and CREDENCE trials, outcomes by frailty status were stratified into BMI categories. Consistent with the aforementioned studies, there was a significant interaction between frailty and BMI. Notably, approximately 66% of frail participants had obesity (BMI > 30 kg/m^2^) and 25% presented with overweight (BMI 25–29.99 kg/m^2^), whereas only 0.2% had a BMI lower than 18.5 kg/m^2^. Collectively, among the 8080 frail patients enrolled across the two trials, merely 15 were classified as underweight [4]. Several meta-analyses have explored the association between BMI and the efficacy outcomes of SGLT2 inhibitors, such as HHF, or cardiovascular and all-cause mortality. Shah et al. reported no significant difference between trials with a mean BMI ≥ 30 kg/m^2^ and < 30 kg/m^2^ (*p* > 0.05) [5]. On the other hand, Zhou et al. found that SGLT2 inhibitors reduced cardiovascular mortality only in patients with obesity, while a reduction in all-cause mortality was observed only in individuals with a BMI ≤ 24.9 kg/m^2^ [57]. However, in both analyses, underweight patients were grouped together with those of normal weight, potentially obscuring specific effects in the lowest BMI category.

Observational studies, although subject to certain biases and limitations, provide real-world data to complement RCTs. For instance, in a cohort of patients with both human immunodeficiency virus (HIV) and HF, low BMI (<18.5 kg/m^2^) was associated with a 57% increase in mortality (HR 1.57; 95% CI 1.03–2.39; *p*  =  0.04) [58]. Furthermore, in the SOLD study, lower baseline BMI increased the risk of SGLT2 inhibitor suspension [13]. In contrast, in a study involving patients with HF and malnutrition, frailty, sarcopenia, or cachexia, SGLT2 inhibitors consistently reduced the incidence of the composite endpoint of death or frailty-related events. Nonetheless, even in this study, 50% of the participants were classified as obese, the mean BMI was 28.7 ± 7.4 kg/m^2^, and only 9.6% patients were cachectic [59].

### 3.3. Lack of Evidence in Institutionalized Patients

Last, but not least, there is an urgent need for studies specifically including institutionalized patients. To the authors’ knowledge, there are currently no trials analyzing the efficacy and safety of SGLT2 inhibitors in this population. In an observational study that analyzed data from 2016, among 102, 451 US nursing home residents treated with antidiabetic medications, only 569 received an SGLT2 inhibitor, and just 27 were treated with it as monotherapy [60]. Hume et al. attributed this low prescribing rate to concerns about volume depletion, urinary tract infections, and medication cost. However, nearly a decade has passed since that study, and much has changed in terms of clinical practice and available evidence, highlighting the urgent need for updated research in this population.

## 4. Conclusions

**Figure 1:**
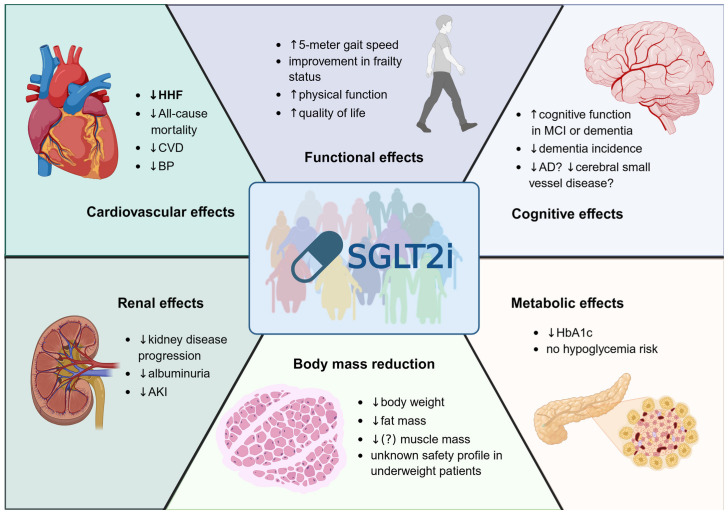
Efficacy of SGLT2 inhibitors in older adults. Abbreviations: ↑, improved; ↓, reduced; SGLT2, sodium-glucose cotransporter 2; HHF, hospitalization for heart failure; CVD, cardiovascular death; BP, blood pressure; MCI, mild cognitive impairment; AD, Alzheimer’s disease; AKI, acute kidney injury; HbA1c, glycated hemoglobin.

Overall, the efficacy of SGLT2 inhibitors in older and frail adults appears to be comparable to that observed in younger populations, with some evidence suggesting even greater absolute benefits due to higher baseline cardiovascular and renal risk. These benefits are particularly pronounced among patients with HF and CKD, in whom SGLT2 inhibitors consistently reduced cardiovascular risk across studies. A particularly uniform finding is the risk reduction in hospitalization for HF, a clinically meaningful outcome for older adults. Each hospitalization may lead to the loss of functional independence, increased risk of infections, decline in physical and cognitive function, and other severe complications. SGLT2 inhibitors do not increase the risk of hypoglycemia, a potentially fatal complication of the treatment of T2DM. In terms of kidney outcomes, older individuals may derive meaningful benefits not only in terms of glomerular filtration but also through reduction in albuminuria and the risk of acute kidney injury. Figure 1 presents a summary of efficacy outcomes in older adults, including cardiorenal risk and domains such as functional status and cognitive health.

However, there are several remaining important evidence gaps. Current trials largely exclude or underrepresent patients with advanced frailty. This was confirmed in the post hoc analyses on frailty, where retrospectively assessed frailty indices indicated that the individuals with the highest level of frailty were rarely included. Consequently, it remains uncertain whether the demonstrated benefits can be generalized to this subgroup. In addition, the efficacy of SGLT2 inhibitors in malnourished older adults has not been adequately explored. As Sinclair et al. have suggested, patients with the malnourished–anorexic phenotype of frailty may not experience the same degree of clinical benefit as sarcopenic–obese frail patients. Further dedicated studies are needed to clarify the risk–benefit profile of SGLT2 inhibitors across the full spectrum of frailty and nutritional status.

## Figures and Tables

**Table 1:** Meta-analyses describing SGLT2 inhibitors’ efficacy in older and frail patients.

	Studies Searched	No. of Studies/Patients	Population	Metabolic Outcomes	Cardiovascular Outcomes	Renal Outcomes
Shah SA et al. [5]	Large (>1000 participants) RCTs, placebo or active control.	8/32, 541	T2DM/HF/CKD ≥65 years; Subgroup analyses (65–74 and ≥75 years)		**All-cause mortality**: RR 0.88; 95% CI 0.83–0.95; *p* < 0.01. **CVD**: RR 0.82; 95% CI 0.74–0.92; *p* <0.01. **HHF**: RR 0.72; 95% CI 0.66–0.79; *p* < 0.01. **MACE**: RR 0.87; 95% CI 0.77–0.99; *p* = 0.04.	**Cardiorenal composite events**: RR 0.77; 95% CI 0.70–0.85; *p* < 0.01.
Hanlon P et al. ^a^ [6]	RCTs, placebo-controlled or active comparator.	172 (8 ^b^)/309, 503 (168, 489 ^b^)	T2DM ≥18 years	Lower **HbA1c** absolute reduction in older patients −0.24%; 95% CI 0.10–0.38; less HbA1c lowering per 30-year higher age for monotherapy.	Greater relative reduction in **MACE** in older patients (HR 0.76; 95% CI 0.62–0.93 per 30 year increment in age).	
Wightman H et al. ^a^ [2]	RCTs, placebo-controlled or active comparator.	10/25, 208	T2DM ≥18 years	**HbA1c**: MD −1.2%; 95% CI −1.4% to −1.0% Slightly attenuated HbA1c reduction with increasing frailty (0.08% [0.02–0.14%, *p* = 0.029] smaller reduction per 0.1-point increase in the FI).		
Aldafas R et al. [7]	RCTs, observational studies, placebo-controlled and active comparator.	20/77, 083	T2DM+HF ≥65 years of frail	HbA1c: MD −0.13, 95% CI −0.41 to 0.14, *p* = 0.34 (SGLT2i vs. non-SGLT2i). After excluding one study with low-dose canagliflozin: MD−0.24, 95% CI −0.43 to −0.06, *p* = 0.009.	**All-cause mortality**: RR 0.81; 95% CI 0.69–0.95, *p* = 0.008. **CVD**: RR 0.80; 95% CI 0.69–0.94, *p* = 0.006. **HHF**: RR 0.69; 95% CI 0.59–0.81, *p*< 0.001.	Composite renal endpoint: RR 0.88; 95% CI 0.65–1.13. AKI: RR 0.92; 95% CI 0.29–2.91
Yamashita I et al. [8]	Large (>1000 participants), placebo-controlled RCTs.	11/79, 370	T2DM/HF/CKD <65 vs. ≥65		MACE: HR: 0.87; 95% CI 0.75–1.01 in older group, without significant subgroup differences (*p* = 0.23). **Composite outcome of CVD or HF exacerbation**: HR: 0.76; 95% CI 0.71–0.82 in older group, without significant subgroup differences (*p* = 0.96).	
Pan SY et al. [9]	RCTs, placebo-controlled or active comparator.	4/41, 654	T2DM ≥65 years; subgroup analyses (65–74 and ≥75 years)	**HbA1c**: MD −0.37%; 95% CI −0.48 to −0.27. **Body weight**: MD −1.85 kg; 95% CI, −2.42 to −1.27.	**HHF**: RR 0.66; 95% CI 0.57–0.77. MACE: RR 0.92; 95% CI 0.82–1.04. Declining NNT with age.	**Renal composite outcomes**: RR, 0.69; 95% CI 0.53–0.89. **Risk of developing albuminuria**: RR 0.71; 95% CI 0.59–0.85. **AKI**: RR 0.70; 95% CI 0.53–0.92.
Karagiannis T et al. [11]	RCTs.	5/93, 502	T2DM <65 vs. ≥65; subgroup analyses (<75 vs. ≥75 years).		MACE: HR 0.87; 95% CI 0.74–1.01, without significant subgroup differences (*p* = 0.38). **HHF**: HR 0.78; 95% CI 0.66–0.93, without significant subgroup differences (*p* = 0.06). **Stroke**: HR 0.83; 95% CI 0.69–1.00, significant difference between <65 vs. ≥65 years subgroups (*p* = 0.02).	**Composite renal endpoint**: HR 0.57; 95% CI 0.43–0.77.

^a^ Individual participant data used in this analysis, ^b^ no. of studies/participants for MACE outcomes, significant outcomes were bolded. Abbreviations: SGLT2i, sodium-glucose cotransporter 2 inhibitors; RCTs, randomized-controlled trials; T2DM, type 2 diabetes mellitus; HF, heart failure; CKD, chronic kidney disease; HbA1c, glycated hemoglobin; MD, mean difference; CI, confidence interval; FI, frailty index; RR, risk ratio; HR, hazard ratio; CVD, cardiovascular death; HHF, hospitalization for heart failure; MACE, major adverse cardiovascular events; NNT, number needed to treat; AKI, acute kidney injury.

**Table 2:** Post hoc analyses of SGLT2 inhibitor trials assessing frailty.

Trial	Frailty Assessment	Frailty Subgroups	Frailty Prevalence	Major Outcomes
DAPA-HF	32-item FI ^a^	non-frail (FI ≤ 0.21); mild frailty (FI 0.21–0.31); severe frailty (FI ≥ 0.31).	not frail, *n* = 2392 (50.4%); more frail, *n* = 1606 (33.9%); most frail, *n* = 744 (15.7%).	**Worsening HF/CVD**: HR = 0.72 (95% CI 0.59–0.89) in non-frail vs. HR = 0.77 (95% CI 0.62–0.97) in moderately frail vs. HR = 0.71 (95% CI 0.54–0.93) in most frail.
DELIVER	30-item FI ^a^	non-frail (FI ≤ 0.21); mild frailty (FI 0.21–0.31); severe frailty (FI ≥ 0.31).	not frail, *n* = 2354 (37.6%); more frail, *n* = 2413 (38.6%); most frail, *n* = 1491 (23.8%).	**Worsening HF/CVD**: HR = 0.85 (95% CI 0.68–1.06) in non-frail vs. HR = 0.89 (95% CI 0.74–1.08) in moderately frail vs. HR = 0.74 (95% CI 0.61–0.91) in most frail. **Placebo-corrected change in KCCQ OSS**: non-frail: +0.3 (95% CI −0.9 to 1.4) vs. moderately frail: +1.5 (95% CI 0.3 to 2.7) vs. most frail: +3.4 (95% CI 1.7 to 5.1) *p* = 0.021.
EMPEROR-Preserved	44-item FI ^a^	non-frail (FI ≤ 0.21); mild frailty (FI 0.21 to <0.30); moderate frailty (FI 0.30 to <0.40); severe frailty (FI ≥ 0.40).	non-frail *n* = 1514 (25.3%); mild frailty *n* = 2100 (35.1%); moderate frailty *n* = 1501 (25.1%); severe frailty *n* = 873 (14.6%).	**CVD/HHF**: HR = 0.59 [95% CI 0.42–0.83], 0.79 [0.61–1.01], 0.77 [0.61–0.96] and 0.90 [0.69–1.16] in non-frail to severe frailty categories. **Likelihood of being in a lower FI category**: OR = 1.12 (95% CI 1.01–1.24) at Week 12 (*p* = 0.030), OR = 1.21 (95% CI 1.09–1.34) at Week 32 (*p ≤* 0.001) vs. OR = 1.20 (95% CI 1.09–1.33) at Week 52, (*p* < 0.001).
EMPA- KIDNEY	Model predicting hospitalization risk at baseline as clinical frailty proxy		Predicted risk of hospitalization during follow-up: ≤20%, *n* = 1988; >20% to ≤35%, *n* = 2504; >35% to ≤45%, *n* = 968; >45% *n* = 1149.	**Kidney disease progression/CVD**: HR = 0.77 (0.56–1.06), vs. HR = 0.65 (0.53–0.80), HR = 0.66 (0.49–0.90), vs. HR = 0.79 (0.62–1.00) in consecutive subgroups.
CANVAS/ CREDENCE	27-item FI ^a^	non-frail (FI ≤ 0.25), frail (FI > 0.25).	non-frail, *n* = 6463 (44%); frail, *n* = 8080 (56%).	**MACE**: HR = 0.80 (95% CI 0.70–0.90) in frail vs. HR = 0.91 (95% CI 0.75–1.09) in non-frail. **CVD**: HR = 0.79 (95% CI 0.67–0.95) in frail vs. HR = 0.94 (95% CI 0.70–1.27) in non-frail. **All-cause mortality**: HR = 0.81 (95% CI 0.70–0.94) in frail vs. HR = 0.93 (95% CI 0.74–1.16) in non-frail.

^a^ Using the Rockwood cumulative deficit approach. Abbreviations: FI, frailty index; HR, hazard ratio; CI, confidence interval; HF, heart failure; CVD, cardiovascular death; KCCQ OSS, Kansas City Cardiomyopathy Questionnaire Overall Summary Score; HHF, hospitalization for heart failure; OR, odds ratio; MACE, major adverse cardiovascular outcome.

**Table 3:** Demographic structure of selected SGLT2 inhibitor trials [15,20,26,49,50,51,52,53,54,55].

Study	Age Group	Total; No. (%)	Active/Placebo; No.
EMPA-REG OUTCOME	<65 years	3893 (55%)	2596/1297
	65–74 years	2475 (35%)	1667/808
	≥75 years	652 (9%)	424/228
CANVAS Program	no information		
DECLARE-TIMI 58	<65 years	9253 (54%)	4631/4622
	65–74 years	6811 (40%)	3413/3398
	≥75 years	1096 (6%)	538/558
CREDENCE	<60 years	1475 (34%)	731/744
	60–69 years	1854 (42%)	950/904
	≥70 years	1072 (24%)	521/551
DAPA-HF	<65 years	1878 (40%)	952/926
	65–74 years	1717 (36%)	830/887
	≥75 years	1149 (24%)	591/558
VERTIS CV	<65 years	4093 (50%)	2718 ^a^/1375
	65–74 years	3242 (39%)	2182 ^a^/1060
	≥75 years	903 (11%)	593 ^a^/310
DAPA-CKD	<60 years	1605 (37%)	807/798
	60–69 years	1499 (35%)	748/751
	70–79 years	997 (23%)	499/498
	≥80 years	197 (5%)	95/102
EMPEROR-Reduced	<65 years	1415 (38%)	675/740
	65–74 years	1316 (35%)	685/631
	≥75 years	999 (27%)	503/496
EMPEROR-Preserved	<65 years	1198 (20%)	593/605
	65–74 years	2213 (37%)	1121/1092
	75–79 years	1275 (21%)	662/613
	≥80 years	1298 (22%)	619/679
DELIVER	<65 years	1345 (21%)	668/677
	65–74 years	2326 (37%)	1136/1190
	≥75 years	2592 (41%)	1327/1265
EMPA-KIDNEY	<60 years	2252 (34%)	1136/1116
	60–69 years	1720 (26%)	853/867
	≥70 years	2637 (40%)	1315/1322
DAPA-TAVI	<80 years	340 (28%)	167/173
	≥80 years	882 (72%)	438/444

^a^ Ertugliflozin 5 mg and 15 mg combined.

## Data Availability

No new data were created or analyzed in this study.
